# Genetic‐Genomic Replication to Identify Candidate Mouse Atherosclerosis Modifier Genes

**DOI:** 10.1161/JAHA.112.005421

**Published:** 2013-02-22

**Authors:** Jeffrey Hsu, Jonathan D. Smith

**Affiliations:** 1 Department of Molecular Medicine, Cleveland Clinic, Lerner College of Medicine, Case Western Reserve University, Cleveland, OH (J.H., J.D.S.); 2 Department of Cellular and Molecular Medicine, Cleveland Clinic, Lerner Research Institute, Cleveland, OH (J.H., J.D.S.)

**Keywords:** atherosclerosis, genetics, transcriptomics

## Abstract

**Objective:**

Genetics plays a large role in atherosclerosis susceptibility in humans and mice. We attempted to confirm previously determined mouse atherosclerosis‐associated loci and use bioinformatics and transcriptomics to create a catalog of candidate atherosclerosis modifier genes at these loci.

**Methods and Results:**

A strain intercross was performed between AKR and DBA/2 mice on the apoE^−/−^ background generating 166 F_2_ progeny. Using the phenotype log10 of the aortic root lesion area, we identified 3 suggestive atherosclerosis quantitative trait loci (Ath QTLs). When combined with our prior strain intercross, we confirmed 3 significant Ath QTLs on chromosomes 2, 15, and 17, with combined logarithm of odds scores of 5.9, 5.3, and 5.6, respectively, which each met the genome‐wide 5% false discovery rate threshold. We identified all of the protein coding differences between these 2 mouse strains within the Ath QTL intervals. Microarray gene expression profiling was performed on macrophages and endothelial cells from this intercross to identify expression QTLs (eQTLs), the loci that are associated with variation in the expression levels of specific transcripts. Cross tissue eQTLs and macrophage eQTLs that replicated from a prior strain intercross were identified. These bioinformatic and eQTL analyses produced a comprehensive list of candidate genes that may be responsible for the Ath QTLs.

**Conclusions:**

Replication studies for clinical traits as well as gene expression traits are worthwhile in identifying true versus false genetic associations. We have replicated 3 loci on mouse chromosomes 2, 15, and 17 that are associated with atherosclerosis. We have also identified protein coding differences and multiple replicated eQTLs, which may be useful in the identification of atherosclerosis modifier genes.

## Introduction

Atherosclerosis is a complex disease with both environmental and genetic susceptibility components. The heritability of atherosclerotic coronary artery disease (CAD) in humans is evident from family history being a significant risk factor.^1–2^ In addition, genome‐wide association studies (GWAS) have identified multiple loci associated with CAD.^3^ However, these studies have a tremendous statistical burden to overcome to meet the threshold of genome‐wide significance, and thus much of the genetic contribution may underreported. Also, GWAS do not ascertain rare variants, and it is becoming increasingly clear that rare variants in aggregate can account for a significant portion of population variance for complex traits such as plasma triglycerides.^4^ Thus, there is still impetus to identify novel genes and pathways that play a role in atherosclerosis susceptibility. Genetics also plays a role in lesion development in mouse models of atherosclerosis, as different inbred strains have markedly different aortic lesion areas.^5^ Mouse models provide an opportunity to tease out the potential genetic modifiers for multigenic phenotypes. We have previously shown that AKR apoE^−/−^ mice have ≈10‐fold smaller aortic root lesions compared with DBA/2 apoE^−/−^ mice when fed a chow diet.^5^ A previous intercross between these 2 strains identified 2 significant and 4 suggestive quantitative trait loci (QTLs) for aortic root lesion area. Just as lesion area is a quantitative trait that can be used for gene mapping studies, gene expression levels can likewise be treated as a quantitative trait to map the expression QTLs (eQTLs), or the loci that control the expression of specific transcripts.^6^ We had previously performed an eQTL analysis using macrophages from the F2 cohort of the AKR apoE^−/−^×DBA/2 apoE^−/−^ strain intercross.^7^ Here, we report atherosclerosis (Ath) QTL and eQTL findings from a second independent strain intercross of these same 2 strains.

We found that both significant Ath QTLs in the prior cross were replicated in the new cross, whereas only one of the prior suggestive Ath QTLs was replicated. We carefully excluded analysis of transcriptome data from microarray probes that contained strain‐specific sequence polymorphisms, and we still found robust replication of macrophage *cis*‐acting eQTLs between the prior and new crosses. We also observed many *cis*‐eQTLs that were conserved between macrophages and endothelial cells (ECs). However, *trans*‐acting eQTLs were not well replicated between the 2 crosses, leading us to believe that there is a high false‐positive rate for the identification of *trans*‐eQTLs. We compiled the lists of all protein coding differences between the AKR and DBA/2 strains, as well as the eQTLs, within the replicated Ath QTLs. These genes provide a comprehensive list of candidates that may be responsible for the observed Ath QTLs.

## Methods

### Mouse and Cell Studies

A DBA/2J apoE^−/−^×AKR/J apoE^−/−^ reciprocal strain intercross was performed to generate an F_1_ cohort and the subsequent F_2_ cohort of 89 males and 77 females. The F_2_ mice were weaned at 21 days and placed on a chow diet. Mice were killed at 16 weeks of age. Femurs were collected from all mice, and the descending aortas were removed from males for culture of ECs as described later. Tail‐tip DNA was prepared from each F_2_ mouse by proteinase K digestion followed by ethanol precipitation. Lesion areas of the aortic root were quantified as previously described.^8^ Genotyping was performed using Illumina Golden Gate mouse genotyping arrays. Genotyping calls were made using Illumina Genome Studio software. In all, 599 informative markers between AKR and DBA/2 were used for QTL and eQTL analyses.

Bone marrow‐derived macrophages (BMMs) were derived as previously described.^7^ To obtain cultured ECs from the F2 mice, descending aortas were isolated, cut into 2‐mm sections, placed on top of matrigel‐coated plates, and grown to confluence (≈10 to 14 days) in DMEM supplemented as previously described.^9^ Cells were treated with dispase and passaged twice before RNA isolation. This protocol was successful in obtaining EC cultures in 48 of 89 attempts. RNA was isolated using Qiagen's total RNA kits and digested with DNase I for 12 minutes at room temperature to remove genomic DNA. RNA integrity was confirmed by agarose gel electrophoresis. cDNA was synthesized using Illumina protocols and reagents and hybridized on Illumina Mouse Ref‐8 v2 microarrays. All expression, phenotype, and genotype data are available in GEO (accession No. GSE35676).

### Statistical Methods

Ath QTLs, using the log10 of aortic root lesion areas, were mapped using the R package qtl (R/qtl).^10^ False discovery rates (FDRs) were estimated with 100 000 permutations using the scanone function in R/qtl. The Ath QTL CIs were calculated within the same software using the Bayesian credible interval function.

Gene expression data were loaded into the R‐package *lumi*^11^ log2 transformed and quantile normalized. eQTLs were mapped using the scanone function in the R/qtl.^10^ Probes were mapped using BLAT^12^ against the mouse mm9 reference genome. Probes that matched to multiple locations or annotated transcripts from Ensembl release 63 were discarded. Probes containing polymorphisms, either an indel or a single nucleotide polymorphism (SNP), or probes mapping to known structural variants between DBA/2 and AKR strains^13–14^ were discarded because they could lead to identification of false *cis*‐eQTLs.^15^ This was performed by taking the genomic locations from BLAT in the University of California Santa Cruz Genome Browser and using Tabix^16^ to retrieve sequence variants from the Mouse Genome Sequencing Project.^13–14^ This filtering resulted in the removal of 2749 probes from the data set.

Human–mouse alignments generated previously by Schwarz et al^17^ were used to obtain the human regions corresponding to our mouse Ath QTLs, and within these regions we identified human GWAS loci^18^ related to CAD.

To compare eQTLs from the current and previous studies,^7^ matches between Affymetrix and Illumina's probes were provided by Illumina (http://www.switchtoi.com/probemapping.ilmn). To perform the combined cross‐QTL analyses, the SNPs from each cross were imput to each other using the fill.geno function in R/qtl, with the simple assumption of no double crossovers. In the prior cross, there were 1947 markers, whereas in the new cross, there were 599 markers, of which only 170 overlapped between the 2, leading to 2376 total markers. There is no metric for imputation quality, but we found that using the original set of markers in the second cross versus the combined set of markers did not greatly alter QTL location or strength. A liberal 20‐Mb window was used between studies to determine if an eQTL overlapped. A combined eQTL analysis was done with data from both studies, using cross membership as an additive covariate and sex as an interactive covariate.

## Results and Discussion

### Atherosclerosis QTL Replication in a New Cross

**Figure 1.**
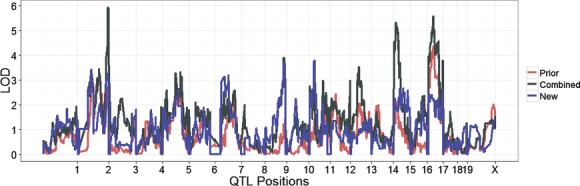
Log10 aortic root lesion atherosclerosis (Ath) quantitative trait locus (QTL) plots. The pink and blue lines show the logarithm of odds (LOD) plots for the prior and new crosses of AKR apoE^−/−^ and DBA/2 apoE^−/−^ mice, respectively. The black line shows the LOD plot for the combined analysis using cross as an additive covariate. In all analyses, sex was used as an interactive covariate.

F_2_ mice were generated using a reciprocal cross strategy from apoE^−/−^ mice on the AKR and DBA/2 backgrounds. Lesion areas in the aortic root were quantified in 166 F_2_ mice (77 female and 89 male). A genome scan was performed for each F_2_ mouse using 599 informative SNPs. We defined significant QTLs as those that have genome‐wide FDRs of <10%, by permutation analysis. Combining both sexes and using sex as an interactive covariate, we identified 3 suggestive Ath QTLs at a logarithm of odds (LOD) score threshold of 2.0 on chromosomes 2, 15, and 17 with FDRs of 15%, 30%, and 16%, respectively (Table 1). Although these Ath QTLs only met the suggestive threshold due to small sample size, each of these Ath QTLs were detected in a prior AKR×DBA/2 strain intercross^19^: *Ath28,* a suggestive QTL on chromosome 2; *Ath22,* a significant QTL on chromosome 15; and *Ath26,* a significant QTL on chromosome 17. We performed a combined Ath QTL analysis using both crosses with cohort membership as an additive covariate and sex as an interactive covariate. As the platform and markers used to genotype the 2 crosses differed, R/qtl^20^ was used to impute the genotypes between the 2 crosses. On chromosome 2, *Ath28* was replicated and had a combined LOD score of 5.9; on chromosome 15, *Ath22* was replicated with a combined LOD score of 5.3; and on chromosome 17, *Ath26* was replicated with a combined LOD score of 5.6 (Figure 1). All of these combined LOD scores met the genome‐wide FDR threshold of <10%, and in fact they all had <5% FDR. However, the suggestive Ath QTLs identified in the first cross using sex as an interactive covariate on chromosomes 5, 3, and 13^19^ were not replicated in the second cross. The approximate 95% Bayesian credible interval was obtained for all 3 loci (Table 1). Thus, the clinical trait mouse QTL model is partially reproducible for a phenotype as complex as lesion area, which has a fairly large coefficient of variation within inbred strains.

**Table 1.** Aortic Root Lesion (log10) QTLs in DBA/2×AKR F2 Cohort

Symbol	Chromosome	Bayesian CI,* Mb	Second Cross LOD Score	Prior Cross LOD Score	FDR	Combined Cross LOD	FDR
*Ath28*	2	165.1 to 179.3	2.8	3.2	0.15	5.9	<0.05
*Ath22*	15	3.6 to 31.9	2.0	2.1	0.30	5.3	<0.05
*Ath26*	17	12.4 to 64.3	2.7	4.4	0.16	5.6	<0.05

QTL indicates quantitative trait loci; LOD, logarithm of odds; FDR, false discovery rate.

* Based on the combined cross‐analysis.

We identified the human chromosome segments orthologous to these mouse loci. We examined whether these human orthologous regions contained common genetic variants associated with CAD, myocardial infarction, or related risk factors by searching the National Human Genome Research Institute GWAS catalog^18^ (Table 2). The known atherosclerosis‐related risk factors included blood lipid levels, subclinical atherosclerosis, type 2 diabetes, and hypertension. There were no human CAD GWAS hits in the region in synteny with *Ath28*, although ≈21% of the *Ath28* interval on chromosome 2 displayed no synteny due to complex expansion in the mouse genome after species divergence. The *Ath22* locus on chromosome 15 contains the corresponding segment on human chromosome 5 that has been associated with subclinical atherosclerosis.^21^ The *Ath26* locus on chromosome 17 corresponds to human chromosomes 6 (primarily) and 19, including the major histocompatibility complex locus, and overlaps with multiple human GWAS loci for CAD and related risk factors (Table 2).

**Table 2.** Corresponding GWAS Hits in Human Orthologous Regions

Chromosome	Position	rsID	Author	Trait	Nearest Gene
2 (*Ath28)*					
No hits					
15 (*Ath22*)					
5	13764419	rs2896103	C.J. O'Donnell	Subclinical atherosclerosis traits (other)	*DNAH5*
5	13769974	rs7715811	C.J. O'Donnell	Subclinical atherosclerosis traits (other)	*DNAH5*
5	13779743	rs1502050	C.J. O'Donnell	Subclinical atherosclerosis traits (other)	*DNAH5*
17 (*Ath26*)					
6	160578859	rs1564348	T.M. Teslovich	LDL cholesterol	*LPA*
6	160578859	rs1564348	T.M. Teslovich	Cholesterol, total	*LPA*
6	160741621	rs3120139	Q. Qi	Lp(a) levels	*SLC22A3*
6	160863531	rs2048327	D.A. Tregouet	Coronary heart disease	*SLC22A3,LPAL2,LPA*
6	160863531	rs2048327	D.A. Tregouet	Coronary heart disease	*SLC22A3,LPAL2,LPA*
6	160907133	rs3127599	D.A. Tregouet	Coronary heart disease	*SLC22A3,LPAL2,LPA*
6	160907133	rs3127599	D.A. Tregouet	Coronary heart disease	*SLC22A3,LPAL2,LPA*
6	160910516	rs12214416	Q. Qi	Lp(a) levels	*LPAL2*
6	160960358	rs6919346	C. Ober	Lp(a) levels	*LPA*
6	160961136	rs3798220	H. Schunkert	Coronary heart disease	*LPA*
6	160962502	rs7767084	D.A. Tregouet	Coronary heart disease	*SLC22A3,LPAL2,LPA*
6	160962502	rs7767084	D.A. Tregouet	Coronary heart disease	*SLC22A3,LPAL2,LPA*
6	160969737	rs10755578	D.A. Tregouet	Coronary heart disease	*SLC22A3,LPAL2,LPA*
6	160969737	rs10755578	D.A. Tregouet	Coronary heart disease	*SLC22A3,LPAL2,LPA*
6	161010117	rs10455872	D.I. Chasman	Response to statin therapy (LDL cholesterol)	*LPA*
6	161010117	rs10455872	Q. Qi	Lp(a) levels	*LPA*
6	161089816	rs1084651	T.M. Teslovich	HDL cholesterol	*LPA*
6	161137989	rs783147	Q. Qi	Lp(a) levels	*PLG*
6	34546560	rs2814982	T.M. Teslovich	Cholesterol, total	*C6orf106*
6	34552797	rs2814944	T.M. Teslovich	HDL cholesterol	*C6orf106*
6	35034800	rs17609940	H. Schunkert	Coronary heart disease	*ANKS1A*
19	8433196	rs7255436	T.M. Teslovich	HDL cholesterol	*ANGPTL4*
19	8469738	rs2967605	S. Kathiresan	HDL cholesterol	*ANGPTL4*
6	31184196	rs3869109	R.W. Davies	Coronary heart disease	*HCG27, HLA*‐*C*
6	32412435	rs3177928	T.M. Teslovich	LDL cholesterol	*HLA*
6	32412435	rs3177928	T.M. Teslovich	Cholesterol, total	*HLA*
6	32669373	rs11752643	F. Takeuchi	Coronary heart disease	*HLA, DRB*‐*DQB*
6	33143948	rs2254287	C.J. Willer	LDL cholesterol	*B3GALT4*
6	43758873	rs6905288	R.W. Davies	Coronary heart disease	*VEGFA*

GWAS indicates genome wide association studies; Lp(a), lipoprotein(a); LDL, low‐density lipoprotein; HDL, high‐density lipoprotein.

### Protein Coding Differences Between AKR and DBA/2 Mice Residing in Ath QTLs

We used a variety of bioinformatic and genomic methods to identify candidate genes that may be responsible for the 3 replicated Ath QTLs. Using the mouse sequence data from 15 common inbred strains,^13–14^ we identified all of the nonsynonymous protein changes in these 3 loci. These strain variable genes on chromosomes 2 (11 genes), 15 (23 genes), and 17 (258 genes) are listed in Table S1, with many genes having >1 amino acid substitution between these 2 strains. We identified many more strain variant genes for *Ath26* on chromosome 17, because it is a very large 52‐Mb gene‐dense interval that contains the highly polymorphic mouse H2 major histocompatibility region. After exclusion of the major histocompatibility genes, we used Polyphen 2^22^ to ascertain in silico the likelihood that each protein coding change would impair protein function, and we found numerous potential protein functional differences between the 2 strains (Tables 3 and S1).

**Table 3.** AKR and DBA/2 Protein Differences Within the *Ath28*,* Ath22*, and *Ath26* CIs Predicted by Polyphen to Be Detrimental

Chromosome	Position	Reference Allele	Alternate Allele	AKR*	DBA*	Gene Symbol	AA Position	AA Alteration	Polyphen Score*
2	165177862	C	T	1/1	0/0	*Zfp663*	646	G>R	0.98
2	165179205	G	A	1/1	0/0	*Zfp663*	198	T>I	0.969
2	172377285	T	C	0/0	1/1	*Tcfap2c*	143	Y>H	0.917
2	173034738	G	C	1/1	0/0	*Zbp1*	280	T>S	0.944
15	3929418	C	T	0/0	1/1	*Fbxo4*	3	G>R	1
15	4705267	G	A	1/1	0/0	*C6*	205	R>K	1
15	4741106	G	T	1/1	0/0	*C6*	533	R>M	0.591
15	4888423	G	T	1/1	0/0	*Heatr7b2*	981	Q>H	0.989
15	4984193	G	A	1/1	0/0	*C7*	242	T>M	0.78
15	9297067	T	G	1/1	0/0	*Ugt3a2*	380	H>Q	0.966
15	11834935	C	T	1/1	0/0	*Npr3*	154	A>T	1
15	27492564	C	T	0/0	1/1	*Ank*	201	A>V	0.932
15	28275570	C	T	0/0	1/1	*Dnahc5*	2434	R>W	0.908
17	12243519	G	C	0/0	1/1	*Park2*	397	E>Q	1
17	17987504	C	T	0/0	1/1	*Has1*	40	A>T	0.638
17	18048331	T	G	1/1	0/0	*Gm7535*	194	D>A	0.958
17	18048648	C	T	1/1	0/0	*Gm7535*	88	M>I	0.875
17	18395099	A	G	1/1	0/0	*Vmn2r94*	117	S>P	1
17	18719527	C	T	0/0	1/1	*Vmn2r96*	53	A>V	1
17	19084957	G	T	1/1	0/0	*Vmn2r97*	836	L>F	0.951
17	19203418	G	T	0/0	1/1	*Vmn2r98*	405	A>S	0.937
17	19204356	G	A	0/0	1/1	*Vmn2r98*	496	E>K	0.979
17	19814743	C	G	1/1	0/0	*Vmn2r102*	352	P>R	0.806
17	19910406	G	C	1/1	0/0	*Vmn2r103*	27	C>S	1
17	19949143	G	T	0/0	1/1	*Vmn2r103*	738	K>N	1
17	20405284	C	T	0/0	1/1	*Vmn2r106*	606	V>I	0.986
17	20415678	A	C	0/0	1/1	*Vmn2r106*	312	Y>D	0.975
17	20608324	C	T	1/1	0/0	*Vmn2r108*	300	M>I	0.793
17	20639402	T	G	1/1	0/0	*Vmn1r225*	47	L>R	0.998
17	21050607	A	T	0/0	1/1	*Vmn1r232*	232	F>I	0.774
17	21423632	C	T	0/0	1/1	*Vmn1r236*	16	T>I	0.953
17	23496190	G	A,T	0/0	1/1	*Vmn2r115*	557	V>F	0.892
17	23802717	A	T	0/0	1/1	*Ccdc64b*	170	T>S	0.961
17	24558772	G	A	1/1	0/0	*Rnps1*	165	G>E	0.953
17	24701163	A	G	1/1	0/0	*Pkd1*	95	E>G	0.985
17	25240572	C	T	1/1	0/0	*Telo2*	664	V>M	0.946
17	25242093	C	T	0/0	1/1	*Telo2*	531	R>H	1
17	25247204	G	A	0/0	1/1	*Telo2*	301	R>C	0.998
17	25305356	A	T	0/0	1/1	*Ccdc154*	380	Q>L	0.924
17	25305357	G	T	0/0	1/1	*Ccdc154*	380	Q>H	0.996
17	25436769	T	C	0/0	1/1	*Prss34*	260	L>P	0.996
17	25457599	A	G	1/1	0/0	*Prss29*	74	T>A	0.997
17	29018352	G	A	1/1	0/0	*Pnpla1*	416	S>N	0.766
17	29018363	T	A	1/1	0/0	*Pnpla1*	420	L>M	0.94
17	29275100	G	T	1/1	0/0	*Rab44*	86	Q>H	1
17	29296944	C	T	1/1	0/0	*Cpne5*	506	V>M	0.862
17	29994582	C	T	1/1	0/0	*Mdga1*	54	D>N	0.997
17	30814214	C	A,T	0/0	1/1	*Dnahc8*	886	L>M	1
17	31145549	A	T	0/0	1/1	*Umodl1*	1304	I>F	0.933
17	31372493	G	A	1/1	0/0	*Ubash3a*	450	V>M	0.948
17	32838830	C	T	1/1	0/0	*Cyp4f15*	378	S>F	0.827
17	33062306	A	G	1/1	0/0	*Cyp4f13*	453	S>P	0.739
17	33731241	C	T	1/1	0/0	*Myo1f*	658	R>C	0.993
17	34048493	A	G	1/1	0/0	*Daxx*	179	N>S	0.734
17	34057211	C	G	0/0	1/1	*Tapbp*	79	L>V	0.669
17	34069629	C	T	0/0	1/1	*Rgl2*	234	A>V	0.999
17	34072961	G	A	0/0	1/1	*Rgl2*	665	V>I	0.969
17	34102686	A	T	0/0	1/1	*Vps52*	663	S>C	0.996
17	34165995	C	T	1/1	0/0	*Slc39a7*	393	V>M	0.968
17	34195857	G	A	1/1	0/0	*Col11a2*	1044	A>T	0.961
17	34196094	G	A	0/0	1/1	*Col11a2*	1079	V>M	0.996
17	34499618	G	A	0/0	1/1	*Btnl2*	242	E>K	0.904
17	34518474	A	C	0/0	1/1	*Btnl3*	300	K>Q	0.81
17	34597021	A	C	1/1	0/0	*BC051142*	264	Q>P	0.663
17	34645055	G	A,T	2/2	0/0	*Btnl6*	482	A>D	0.996
17	34645142	C	G	0/0	1/1	*Btnl6*	453	S>T	0.936
17	34645839	T	C	0/0	1/1	*Btnl6*	337	Q>R	0.752
17	34652479	C	G	0/0	1/1	*Btnl6*	85	E>Q	0.893
17	34670298	A	G	1/1	0/0	*Btnl7*	426	F>L	0.999
17	34670922	G	A	1/1	0/0	*Btnl7*	334	R>C	0.999
17	34679441	C	T	1/1	0/0	*Btnl7*	132	G>R	0.997
17	34721459	G	A	0/0	1/1	*Notch4*	1469	R>Q	0.997
17	34724653	A	G	1/1	0/0	*Notch4*	1873	Y>C	0.995
17	34735125	G	A	1/1	0/0	*Ager*	31	G>E	1
17	34782359	C	T	0/0	1/1	*Fkbpl*	52	P>L	0.957
17	34787670	G	T	0/0	1/1	*Atf6b*	233	A>S	0.975
17	34808447	A	G	0/0	1/1	*Tnxb*	273	Q>R	0.963
17	34831297	A	C	1/1	0/0	*Tnxb*	1903	E>A	0.944
17	34832575	C	G	1/1	0/0	*Tnxb*	2020	H>D	0.794
17	34833521	C	T	0/0	1/1	*Tnxb*	2167	T>I	0.993
17	34840315	C	T	0/0	1/1	*Tnxb*	2494	P>S	0.992
17	34867829	C	T	0/0	1/1	*C4b*	1442	R>K	0.613
17	35163365	G	A	0/0	1/1	*Ng23*	129	T>M	0.941

AA indicates amino acid; MHC, major histocompatibility complex. Table excludes the MHC genes and several zinc‐finger protein genes.

* 0 is the reference allele; 1 is the first or only alternate allele; and 2 is the second alternate allele.

* Probability of detrimental amino acid substitution, using >0.5 as the threshold.

On chromosome 2, only 3 protein changes were predicted to be damaging in 1 strain relative to the other strain. *Zbp1* is a Z‐DNA binding protein, *Tcfap2c* is an AP‐2 transcription factor involved in early development, and Zfp663 is a zinc‐finger protein; none of these proteins have been previously implicated in atherosclerosis susceptibility. On chromosome 15, there were several protein coding changes that are predicted to be detrimental in 1 strain versus the other. Two components of the complement system, *C6* and *C7,* have predicted functional differences, both with the AKR version being detrimental. The complement system has potential roles in cardiovascular disease as previously reviewed.^23^ On chromosome 17, there were 50 genes with predicted detrimental changes, and many additional changes in major histocompatibility complex genes, that were not subjected to the Polyphen analysis. Some notable protein changes were found in *Rab44*,* Collagen 11a2*, and *Notch4*, which can alter cellular vesicular trafficking, extracellular matrix, and signal transduction, respectively, all potential atherosclerosis modifiers.

### eQTLs in Bone Marrow–Derived Macrophages and Endothelial Cells

The global profile of gene expression in bone marrow–derived macrophages was assayed using Illumina microarrays from 79 female and 81 male F_2_ mice. With the same 599 SNPs used to map the Ath QTLs, we mapped eQTLs, or loci that are associated with the expression of each transcript, using sex as an additive covariate. An eQTL was defined as a *cis*‐eQTL if the eQTL mapped within 20 Mb of the probe position. A *trans*‐eQTL is defined as the QTL mapping anywhere else on the genome. To eliminate spurious eQTLs, we filtered out 2479 expression array probes that contained an SNP or insertion/deletion between these 2 strains, which could lead to altered probe hybridization impairing an accurate measure of gene expression. We validated that these strain variant probes would indeed lead to false *cis*‐eQTLs with on average an LOD score that was double the LOD score of comparable probes containing no variant (LOD 14.7 versus 7.6, *P*<2.2×10^−6^). In addition, the transcripts with the nonreference SNP allele overlapping the probes were overwhelmingly called with lower expression values versus the transcripts containing the reference allele (Figure S1). After filtering out probes that were not expressed above background in ≥25% of the samples, 9600 probes were evaluated for eQTLs. We used a stringent FDR cut‐off of 5% to identify *cis*‐eQTLs, which corresponded to an LOD score threshold of 2.4 and found 937 *cis*‐eQTLs (Table S2). Table 4 shows the top 25 *cis*‐eQTLs ranked by LOD score. Because *trans*‐eQTLs are indirect, and often not as strong as *cis*‐eQTLs, we applied both a liberal FDR cutoff of 30% and a stringent 5% cutoff. The 30% and 5% FDR thresholds corresponded to LOD scores of 2.81 and 3.75, respectively, with 3797 and 551 *trans*‐eQTLs identified, respectively (Table S3). Table 5 shows the top 25 *trans*‐eQTLs ranked by LOD score.

**Table 4.** Top 25 *cis*‐eQTLs by LOD Score in BMMs

Gene Symbol	QTL Marker	QTL Chromosome	QTL Marker Position	LOD
*Rnps1*	rs3719497	17	24100880	55.9
*Fblim1*	rs3688566	4	140026440	54.0
*Zfp277*	rs13481408	12	35475720	44.7
*2210012G02Rik*	rs3709486	4	109968331	43.9
*Atg9b*	CEL‐5_24211033	5	24211033	42.5
*Vill*	rs3669563	9	117891342	41.2
*Gjb4*	gnf04.123.367	4	126400415	35.3
*Gm962*	CEL‐19_5283144	19	5283144	35.0
*Insl6*	rs3090325	19	26007713	33.3
*Gm962*	CEL‐19_5283144	19	5283144	32.8
*Hint2*	CEL‐4_40541402	4	40541402	32.6
*Agpat5*	rs3657963	8	16576750	32.1
*Prss22*	rs3726555	17	15215815	31.5
*Ccdc163*	rs3709486	4	109968331	30.7
*Sys1*	rs3671849	2	163215888	30.4
*Abhd1*	rs13469943	5	29486094	30.1
*Tuft1*	rs13477261	3	92344335	28.0
*Usp2*	rs4135590	9	43060131	28.0
*H2*‐*Gs10*	rs3682923	17	34343989	27.7
*Abhd1*	rs13469943	5	29486094	27.5
*Zfp420*	rs4226520	7	18758740	27.5
*H2‐T10*	rs6298471	17	35059374	26.9
*Pdxdc1*	rs4163196	16	13143895	26.5
*Fgr*	rs3663950	4	134187547	26.1
*Scamp5*	rs13480208	9	55395056	25.7

eQTL indicates expression quantitative trait loci; LOD, logarithm of odds; BMM, bone marrow macrophage.

**Table 5.** Top 25 *trans*‐eQTLs by LOD Score in BMMs

Gene Symbol	QTL Chromosome	QTL Marker Position	QTL Marker	LOD	Probe Chromosome	Probe Location
*Akr1e1*	4	141638718	rs3023025	28.6	13	4592177
*Mcee*	7	51544526	rs3714908	16.5	7	71556822
*Man2a2*	7	65348455	rs13479355	16.4	7	87505769
*Gdpd3*	7	111285662	rs6275579	16.1	7	133914693
*Pde2a*	7	87305015	rs13479427	15.1	7	108661076
*Alg8*	7	80158909	rs4226783	14.7	7	104540400
*Gstp2*	1	188355284	rs3667164	14.2	19	4041930
*Man2a2*	7	66776784	rs13479358	13.0	7	87505788
*Dgcr6*	17	34343989	rs3682923	12.4	16	18070266
*Crym*	7	104533846	rs13479477	11.7	7	127330117
*Iqgap1*	7	65348455	rs13479355	10.8	7	87857712
*Stab 2*	13	63888326	rs4229817	10.7	10	86304140
*Heatr5a*	12	29204179	rs6223000	10.5	12	52977981
*Mlycd*	13	70810674	rs13481880	9.8	8	121934766
*Arap1*	7	77850273	CEL‐7_77850273	9.0	7	108560953
*Ifitm6*	7	126965988	rs3663988	9.0	7	148201750
*Fam168a*	7	77850273	CEL‐7_77850273	8.3	7	107987317
*Ints3*	3	60037290	rs13477138	7.7	3	90195467
*Plscr4*	9	63239299	gnf09.057.223	7.5	9	92387070
*Snrpn*	7	33835314	rs8260975	7.3	7	67128021
*Fam125b*	2	10929543	rs6240512	6.9	2	33585812
*Unc45a*	7	66776784	rs13479358	6.9	7	87470214
*Capn10*	17	45506664	rs6409750	6.6	1	94844286
*Nasp*	4	91308682	rs6271003	6.6	4	116273846

eQTL indicates expression quantitative trait loci; LOD, logarithm of odds; BMM, bone marrow macrophage.

Lusis has proposed that mouse strain effects on EC function may underlie some strain effects on atherosclerosis.^24^ We successfully cultured primary aortic ECs from 48 male F_2_ mice used in the atherosclerosis study and assayed global gene expression by microarray. As expected, these cells expressed high levels of canonical EC transcripts encoding the proteins Tie2, the Vegf receptors, and von Willebrand factor, all of which were lowly expressed in BMMs. We applied the same FDR thresholds as in the macrophage analysis to identify EC *cis*‐ and *trans*‐eQTLs. At the 5% FDR threshold, corresponding to an LOD score of 2.47, we identified 440 *cis*‐eQTLs (Table S4 and top 25 in Table 6). For *trans*‐eQTLs, the 30% and 5% FDR thresholds corresponded to LOD scores of 2.70 and 3.92, with 4894 and 365 *trans*‐eQTLs identified, respectively (Table S5 and top 25 in Table 7).

**Table 6.** Top 25 *cis*‐eQTLs by LOD Score in ECs

Gene Symbol	QTL Chromosome	QTL Marker Position	QTL Marker	LOD
*Thumpd1*	7	107424656	rs3709679	31.7
*Mod1*	9	90513305	gnf09.087.298	29.2
*Mff*	1	85865441	rs3723062	28.0
*Paip1*	13	115056838	rs13482035	27.5
*Ercc5*	1	44668113	CEL‐1_44668113	24.3
*Lrrc57*	2	117074373	rs13476723	24.1
*G430022H21Rik*	3	122002332	rs3659836	23.1
*Aqr*	2	111095530	rs13476698	22.2
*Atpbd*3	7	33574760	rs8255275	21.1
*Abhd1*	5	29486094	rs13469943	18.9
*Scoc*	8	82188194	rs13479863	17.1
*Zfp277*	12	34954150	rs13481406	16.7
*Grwd1*	7	33574760	rs8255275	16.2
*Ugt1a6a*	1	87104170	UT_1_89.100476	16.0
*2610019P18Rik*	5	135836567	rs4225536	15.0
*Rnf41*	10	122911418	rs13480803	15.0
*Slc25a3*	10	93288075	rs13480712	14.9
*Pdxdc1*	16	12215630	rs4162800	14.8
*4930455C21Rik*	16	30855920	rs4168640	14.6
*Il3ra*	14	7401248	rs3150398	14.6
*Tpmt*	13	46624183	rs6411274	14.5
*Arid4b*	13	12961456	rs13481697	14.2
*Pdxdc1*	16	12215630	rs4162800	14.0
*BC031748*	X	129847872	rs13484031	13.0

eQTL indicates expression quantitative trait loci; LOD, logarithm of odds; EC, endothelial cells.

**Table 7.** Top 25 *trans*‐eQTLs by LOD Score in ECs

Gene Symbol	QTL Marker	QTL Chromosome	QTL Marker Position	LOD	Probe Chromosome	Probe Location
*Tob2*	rs13480854	11	7518795	7.24	15	81679613
*Adi1*	rs6344105	12	63022076	6.90	12	29366235
*Mrgprg*	rs13482407	14	114388484	6.46	7	150950410
*Rb1*	rs3663355	4	47358427	6.39	14	73595412
*P2ry6*	rs3707067	7	85992410	6.20	7	108086188
*Slc10a7*	rs13477617	4	27105003	5.99	8	81230781
*Trp53bp1*	rs13482418	15	3498960	5.94	2	121025364
*Mgst3*	rs6279930	1	137268795	5.92	1	169303924
*Fam168a*	rs13479324	7	57878499	5.89	7	107987317
*Eml1*	rs6393948	11	103272243	5.84	12	109726577
*Mrvi1*	rs13476928	2	174162530	5.82	7	118012039
*Tprn*	rs3669563	9	117891342	5.78	2	25125227
*Cdk2ap2*	rs6290836	14	9149500	5.71	19	4098608
*Spcs3*	gnf18.051.412	18	53592447	5.64	8	55606073
*Trap1*	rs3699561	1	130962369	5.54	16	4040058
*Eral1*	gnf04.123.367	4	126400415	5.50	11	77887218
*BC013529*	rs13477617	4	27105003	5.47	10	7487771
*Tcf20*	rs13483085	17	66984514	5.43	15	82640181
*Prpsap2*	rs13482673	15	82560799	5.42	11	61543254
*Rin3*	rs3721056	9	71328971	5.38	12	103628978
*Dhcr24*	rs3664408	2	161443571	5.37	4	106259325
*Nxt1*	rs3663950	4	134187547	5.36	2	148501307
*Rtn2*	rs13476928	2	174162530	5.34	7	19881245
*Mlst8*	rs13482035	13	115056838	5.33	17	24610627

eQTL indicates expression quantitative trait loci; LOD, logarithm of odds; EC, endothelial cells.

**Figure 2.**
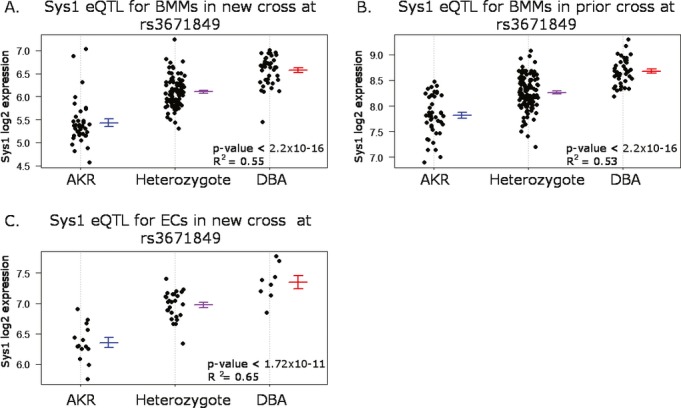
Example of a replicated *cis*‐eQTL between tissues (A and C) and between studies (A and B) of *Sys1*, an integral Golgi‐associated membrane protein. Means±SEM are shown adjacent to the individual values. *P* and *R*^2^ values were obtained by linear regression with sex as an additive covariate. eQTL indicates expression quantitative trait loci; BMM, bone marrow macrophage; EC, endothelial cell.

To evaluate cross‐tissue eQTLs, we counted the number of *cis*‐eQTLs (5% FDR) and *trans*‐eQTLs (30% FDR) that were found in both macrophages and ECs. We identified 156 *cis*‐eQTLs (Table S6) common in both tissues, although our power was limited by the relatively small number of EC samples. We identified 12 cross‐tissue *cis*‐eQTLs that were located in the 3 replicated Ath QTLs in chromosomes 2, 15, and 17 (Table 8). An example of a cross‐tissue *cis*‐eQTL within an Ath QTL interval is *Sys1*, coding for the Golgi‐localized integral membrane protein homolog (Figure 2). In contrast to the 156 cross‐tissue *cis*‐eQTLs, there were only 18 cross‐tissue *trans*‐eQTLs at the 30% FDR threshold that overlapped the 2 tissues (Table 9). A replicated *trans*‐eQTL is defined one in which the *trans*‐eQTL markers map within 10 Mb of each other. On inspection, it appears that 3 of these cross‐tissue *trans*‐eQTLs on chromosome 7 may in fact be *cis*‐eQTLs, because the positions of the gene and the markers were on the chromosome 7 and only slightly greater that the 20‐Mb cutoff used to classify *cis‐*eQTLs. The low number of cross‐tissue *trans*‐eQTLs has been noted in previous studies.^25^ One of these cross‐tissue *trans*‐eQTLs mapped to the *Ath22* interval on chromosome 15, which was associated with the expression of the *Klf2* transcription factor on chromosome 8.

**Table 8.** *cis*‐eQTLs That Are Found in Both ECs and BMMs at <5% FDR That Also Reside Within the *AthQTLs*

Probe_ID	Gene Symbols	QTL Chromosome	BMM eQTL Position	EC eQTL Position	BMM LOD	EC LOD
ILMN_2674425	*Sys1*	2	163215888	163215888	30.45	10.91
ILMN_1216029	*Cstf1*	2	174162530	174162530	3.87	3.67
ILMN_2869312	*Fbxo4*	15	5744460	4222769	7.06	10.81
ILMN_3123120	*Rnps1*	17	24100880	15215815	55.90	11.24
ILMN_2601877	*Brd4*	17	34343989	34343989	2.97	4.60
ILMN_2810539	*H2‐Gs10*	17	34343989	39772541	27.72	9.78
ILMN_2691360	*Mrps10*	17	39772541	43897393	6.98	3.99
ILMN_1256171	*Tmem63b*	17	43897393	43897393	16.68	8.49
ILMN_2734045	*Mrpl14*	17	45506664	35059374	20.28	5.97
ILMN_2804487	*Aif1*	17	45506664	39772541	2.62	3.49
ILMN_2761876	*Fez2*	17	62066360	72668814	3.69	3.25
ILMN_3090123	*Dync2li1*	17	82051202	87503632	22.79	6.35

eQTL indicates expression quantitative trait loci; EC, endothelial cells; BMM, bone marrow macrophage; FDR, false discovery rate; LOD, logarithm of odds.

**Table 9.** *trans*‐eQTLs That Are Found in Both ECs and BMMs at <30% FDR

Probe_ID	Gene Symbols	QTL Chromosome	BMM eQTL Position	EC eQTL Position	BMM LOD	EC LOD	Probe Chromosome	Probe Position
ILMN_2759167	*Gtpbp5*	1	152747565	160294867	4.65	2.98	2	179820490
ILMN_2629375	*1110038F14Rik*	3	41838640	41838640	3.32	4.03	15	76780014
ILMN_2740285	*Fancl*	3	126894715	132189425	2.85	2.86	11	26371341
ILMN_2602837	*Akr1e1*	4	141638718	134187547	28.63	2.82	13	4592177
ILMN_1245307	*Fbln2*	5	39608047	39608047	3.04	2.79	6	91221963
ILMN_2685329	*Hspg2*	5	39608047	39608047	2.87	2.88	4	137126406
ILMN_2622057	*Tsen2*	5	117374791	111603432	3.16	4.78	6	115527976
ILMN_2791578	*Gspt1*	5	131766121	135836567	3.14	2.96	16	11220678
ILMN_1255175	*Unc45a*	7	66776784	65348455	6.09	3.31	7	87470402
ILMN_2893879	*Gdpd3*	7	111285662	112152410	16.13	5.25	7	133914693
ILMN_2689056	*Cd2bp2*	7	112152410	112152410	6.11	5.08	7	134335721
ILMN_2751354	*Pde4dip*	8	84344531	86397354	4.17	3.17	3	97542917
ILMN_2604029	*Klf2*	15	34383985	41359817	3.17	3.10	8	74844875
ILMN_1256434	*Pigk*	15	72483350	72483350	3.08	3.85	3	152448803
ILMN_2836924	*Wdr45*	15	95679367	92664963	2.97	2.74	20	7305117
ILMN_3114998	*Zfp658*	15	95679367	92664963	3.44	3.70	7	50830408
ILMN_2672778	*Abhd1*	17	66984514	69670995	3.11	3.18	5	31255322
ILMN_3038459	*Morf4l1*	19	21112530	29540192	2.86	2.76	9	89998557

eQTL indicates expression quantitative trait loci; LOD, logarithm of odds; EC, endothelial cell; BMM, bone marrow macrophage; FDR, false discovery rate.

### Macrophage eQTL Replication Between Different Crosses and Different Platforms

**Figure 3.**
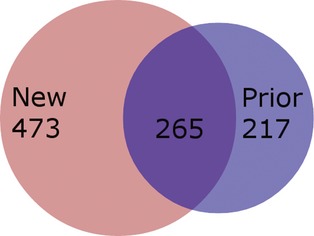
Venn diagram of the overlap between the *cis*‐eQTL in the new cross and the old cross. Transcripts were limited to only the transcripts that were called present in both and had corresponding probe between the platforms. eQTL indicates expression quantitative trait loci.

A macrophage eQTL study was performed in the previous AKR×DBA/2 F_2_ intercross; however, different genetic markers and different expression array platforms were used. To examine replication of macrophage eQTLs between the current and previous study, we reanalyzed the prior data by imputing to the currently used 599 strain‐specific SNPs and mapping the Affymetrix gene expression probes to the currently used Illumina probes. After filtering out probes not mapped to the Illumina platform or those that were excluded in our new cross, only 5678 probes remained for analysis. We then performed the eQTL analysis of the prior dataset using sex as an additive covariate and obtained *cis‐* and *trans‐*eQTLs at the same FDRs as described earlier (summary statistics in Table 10). Of the 738 and 482 *cis*‐eQTLs identified in the prior and new crosses, respectively, 265 were replicated, representing 36% and 55% of the input *cis*‐eQTLs in the old and new cross, respectively (Figure 3, Table S7). The *cis*‐eQTL replication percentage range (36% to 55%) in our study is somewhat lower than that of previously published replication study by van Nas et al that found a *cis*‐eQTL replication rate of ≈50% to 60%.^25^ However, van Nas et al used the same platform and genotyping markers across their 2 studies, whereas we used separate platforms. In addition, van Nas et al probably overestimated the replication rate, because they did not remove probes containing strain polymorphic SNPs as we did in our study. We demonstrated that inclusion of the strain‐polymorphic probes leads to strong false‐positive eQTLs. *Sys1* not only had a cross‐tissue *cis*‐eQTL, but it is also an example of a cross‐study replicated *cis*‐eQTL in BMMs (Figure 2). The SNP rs3671849, within the *Ath28* locus, displayed a strong additive effect on the expression of *Sys1*, with the DBA/2 allele expressed higher. This marker was associated with 51% and 42% of the variation in BMM *Sys1* gene expression in the new and prior crosses, respectively, and 63% of the variation in EC *Sys1* gene expression.

**Table 10.** Summary Statistics and Replication of Bone Marrow Macrophage *cis*‐ and *trans*‐eQTLs for the Prior and New Crosses Using the Restricted Set of Common Probes

	*cis‐*eQTLs	*trans*‐eQTLs
No. of eQTLs (5% FDR) prior cross	738	281
No. of eQTLs (5% FDR) new cross	482	274
No. of eQTLs common between old and new (5%)	265	5
No. of eQTLs common between old and new (30%)	ND	23
No. of eQTLs in combined analysis (5% FDR)*	783	703
No. of eQTLs in combined analysis (30% FDR)*	ND	3158

eQTL indicates expression quantitative trait loci; FDR, false discovery rate; ND, not determined.

* Combined sex eQTL analysis in both crosses using sex as an additive covariate.

**Figure 4.**
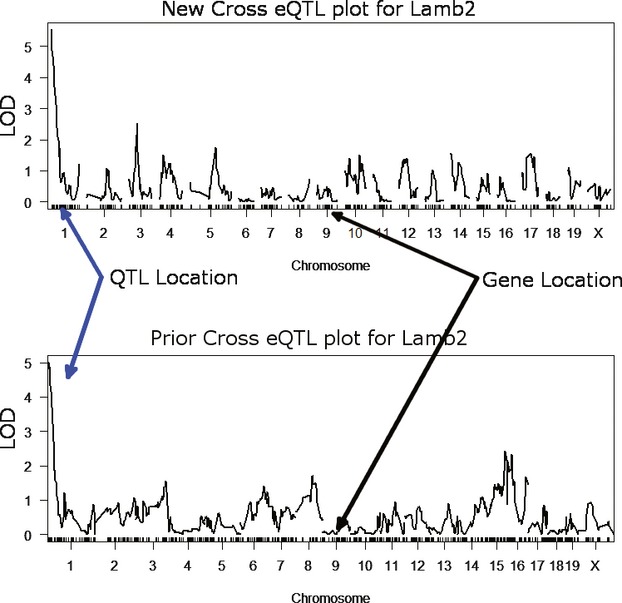
An example of a replicating *trans*‐eQTL on chromosome 4 for the *Lamb2* gene residing on chromosome 9. eQTL indicates expression quantitative trait loci; LOD, logarithm of odds.

There were only 5 *trans*‐eQTLs that replicated between the 2 crosses, or 0.9% and 0.6% of the old cross and new cross *trans*‐eQTLs, respectively, at a 5% FDR LOD score cutoff (Table 11). The LOD plots and allele effects on gene expression for the *Lamb2* gene, which had a replicated *trans*‐eQTL, is shown in Figure 4. Relaxing the FDR to 30% in both crosses resulted in 23 *trans*‐eQTLs that replicated between the studies, or 6% and 4% of the old cross and new cross *trans*‐eQTLs, respectively. This is lower than the ≈19% *trans*‐eQTL replication rate observed by van Nas et al; however, the same caveats apply to our analysis concerning our use of 2 expression array and SNP platforms.^25^

**Table 11.** Replicated *trans*‐eQTL Between Crosses at the 5% FDR Level

Prior Probe ID	New Probe ID	QTL Chromosome	Prior QTL Marker Position, Mb	New QTL Marker Position, Mb	Prior LOD Score	New LOD Score	Gene Symbol	Probe Chromosome	Probe Position, Mb
1416513_at	ILMN_2699488	1	11.3	4.5	5.0	6.4	*Lamb2*	9	108.4
1419423_at	ILMN_2737368	13	74.5	63.9	6.3	10.7	*Stab 2*	10	86.3
1437470_at	ILMN_2780759	1	169.2	188.4	7.1	4.1	*Pknox1*	17	31.7
1448609_at	ILMN_2493175	1	8.1	13.0	13.9	5.1	*Tst*	15	78.2
1451343_at	ILMN_1240149	8	44.5	43.9	5.8	4.1	*Vps36*	8	23.4

eQTL indicates expression quantitative trait loci; LOD, logarithm of odds; FDR, false discovery rate.

As an alternative to examining replication of eQTLs, we combined the data from both F_2_ cohorts and performed a combined analysis of *cis‐* and *trans*‐macrophage eQTLs using sex and cross as additive covariates. The combined method has more power to identify eQTLs than the replication method because it uses a larger sample size and thus is not penalized by a near‐miss false‐negative result in 1 of the 2 crosses. In the combined analysis, there were 783 *cis*‐eQTLs at a 5% FDR threshold (Tables 10 and S8). An example of a significant *cis*‐eQTL found in the combined analysis, but not in the replicated analysis, is an eQTL for *Wdr70*, a WD40 repeat adapter protein. In the combined analysis, there were 160 *cis*‐eQTLs that were found that were not found in either analysis. Furthermore, there were 703 and 3158 *trans*‐eQTLs at the 5% and 30% FDR thresholds in the combined analysis, respectively (Tables 10 and S9).

We systematically searched for replicated eQTLs within the Ath QTL regions to identify potential atherosclerosis modifier candidate genes. In total, there were 14 genes that met this criterion, and for each we determined the correlation of macrophage gene expression and lesion area within the F_2_ mice of the prior and current crosses. Twelve of these correlations had conserved directions in the 2 crosses (Table 12). At the *Ath28* locus on chromosome 2, we identified 2 replicated macrophage *cis*‐eQTLs, of which *Sys1* may have a connection to cholesterol ester metabolism. *Sys1*, whose expression was positively associated with lesion area, is a Golgi‐localized integral membrane protein that is essential for the targeting for several proteins to the Golgi complex and membrane vesicles^26^ including the small GTPases *Arl3p* and *Arfrp1*. Deletion of *Arfrp1* results in loss of lipid droplet formation in adipocytes^27^; lipid droplets in macrophages store cholesterol esters and thus may play an important role in modifying atherosclerosis. At the *Ath26* locus on chromosome 17, there were 9 replicated eQTLs with a shared direction of lesion area correlation, 2 of which have some prior link to atherosclerosis. *Prss22* is a serine protease that converts prourokinase‐type plasminogen activator into its enzymatically active form, abbreviated as uPA.^28^ We found that *Prss22* expression was inversely correlated with atherosclerosis; thus, we would predict that uPA activity may also be inversely correlated with atherosclerosis. However, this is not the case, as previous studies have shown that macrophage expression of uPA is positively associated with atherosclerosis in apoE‐deficient mice.^29–30^ *Ltb*, encoding lymphotoxin‐β (a member of the tumor necrosis factor gene family), resides in the *Ath26* locus, and its expression was positively correlated with lesion area. Lymphotoxin‐β receptor signaling in the arterial media beneath atherosclerotic plauques has been found to promote tertiary lymphoid organogenesis.^31^ In addition, circulating levels of lymphotoxin‐β receptor in humans were positively associated with coronary artery calcium scores.^32^ However, it is difficult to interpret whether these findings are relevant to our observed correlation of macrophage *Ltb* expression and lesion area. None of the other replicated eQTLs at the Ath loci had obvious known connections to pathways implicated in atherosclerosis.

**Table 12.** Replicated *cis*‐eQTL Within Replicated Ath QTL Intervals That Have Replicated Direction of Expression–Lesion Correlation

Illumina Probe ID	Affymetrix Probe ID	Gene Symbol	QTL Chromosome	Expression–Lesion Correlation New Cross*	Expression–Lesion Correlation Old Cross*
ILMN_2674425	1450057_at	*Sys1*	2	0.06	0.18
ILMN_1216029	1448597_at	*Cstf1*	2	−0.16	−0.13
ILMN_2710121	1416441_at	*Pgcp*	15	−0.19	−0.12
ILMN_2688287	1420352_at	*Prss22*	17	−0.04	−0.18
ILMN_2615207	1418321_at	*Eci1*	17	−0.29	−0.15
ILMN_1219908	1418344_at	*Tmem8*	17	0.11	0.18
ILMN_1218891	1419547_at	*Fahd1*	17	−0.06	−0.13
ILMN_2667889	1417173_at	*Atf6b*	17	0.02	0.13
ILMN_1241923	1449537_at	*Msh5*	17	−0.18	−0.26
ILMN_2726308	1449021_at	*Rpp21*	17	−0.26	−0.10
ILMN_1258283	1419135_at	*Ltb*	17	0.16	0.16
ILMN_2761876	1434348_at	*Fez2*	17	−0.08	−0.07

eQTL indicates expression quantitative trait loci; Ath QTL, atherosclerosis quantitative trait loci.

* Pearson's correlation *R* value.

## Conclusions

We found that phenotypic QTLs for the complex trait of atherosclerosis were partially reproducible. Of the 6 Ath QTLs indentified in the prior cross, the 2 significant QTLs replicated, as did 1 of 4 suggestive QTLs in a combined analysis with the new cross. In the new and smaller cross, all 3 of the suggestive Ath QTLs were found in the prior cross. Based on these results, it may be prudent to replicate phenotypic QTLs before embarking on extensive gene discovery and fine mapping studies.

We also report here than many *cis*‐eQTLs can be replicated in independent crosses even when the genotyping and gene expression platforms used differed between the studies. This is not unexpected because the *cis*‐eQTLs are direct and often have very strong effects on gene expression. However, we found a lower rate of *trans*‐eQTL replication compared with another replication study.^25,33^ Our conclusion is that many *trans*‐eQTLs identified in mouse studies may be false positives, or very sensitive to environmental effects, making replication less likely.
